# Extraskeletal mesenchymal chondrosarcoma arising from soft tissues: A rare case report

**DOI:** 10.1002/cnr2.1883

**Published:** 2023-08-09

**Authors:** Razieh Shahnazari, Fatemeh Montazer, Shahriar Shirzadi, Sina Karaji

**Affiliations:** ^1^ Radiology Department of Iran University of Medical Sciences (IUMS) Tehran Iran; ^2^ Firoozabadi Clinical Research Development Unit (FACRDU) Iran University of Medical Sciences (IUMS) Tehran Iran; ^3^ Radiology Department of Hamedan University of Medical Sciences Hamedan University of Medical Sciences Hamedan Iran

**Keywords:** biphasic pattern, extraskeletal, mesenchymal chondrosarcoma, tumor

## Abstract

**Background:**

Chondrosarcomas are an exceedingly rare form of cancer, impacting only a few individuals per million. Among chondrosarcomas, a small fraction belongs to the mesenchymal sub‐type. Furthermore, only one‐third of mesenchymal chondrosarcomas manifest in extraskeletal locations.

**Case:**

A 38‐year‐old woman was referred by a midwife after experiencing pain in the right upper quadrant of her right breast for 2 months. The mass had been palpable for 1 week before the initial assessment. According to radiological evaluations, the tumor is outside breast tissue and not connected to the bones. Hence, a biopsy of the mass is done. The biphasic morphology of the tumor during pathological evaluation, in addition to immunohistochemistry testing, confirms the diagnosis of extraskeletal mesenchymal chondrosarcoma (EMCS). Finally, the mass was surgically removed, and 6 months of chemotherapy were administered to the patient.

**Conclusion:**

Given the tumor's rarity and the lack of established guidelines, diagnosing EMCS can be challenging and prone to errors. As such, meticulous sampling, along with precise pathological and imaging investigations, is imperative to accurately establish the diagnosis of these tumors.

## 1 INTRODUCTION

Chondrosarcoma (CS) represents a category of primary malignant bone tumors characterized by the production of mixed, slow‐growing neoplastic tissue composed of hyaline cartilage. Remarkably, CS stands as the second‐most frequent primary bone cancer.^1^ Mesenchymal chondrosarcoma (MCS) constitutes approximately 2%–4% of all CSs and is relatively infrequent in extraskeletal locations.^2^, ^3^, ^4^ MCS exhibits a slight male predominance and has the potential to arise at any age. However, it is more commonly observed in the second and third decades of life compared to other CSs, which tend to manifest at an earlier stage in life. Notably, approximately one‐third of all MCS cases involve extraskeletal soft tissues, contributing to the uniqueness and diverse presentations of this rare tumor type.^5^, ^6^, ^7^ Indeed, young adults are more predisposed to developing extraskeletal mesenchymal chondrosarcoma (EMCS), and this particular variant is associated with a heightened risk of distant metastasis. Although EMCS can potentially occur anywhere with mesenchymal cells, it predominantly manifests in specific locations, including the orbit, leptomeninges, and lower extremities, with a notable predilection for the thigh region.^8^, ^9^

Despite the absence of a distinctive radiological appearance, a CT scan of the patient can offer valuable insights. It may reveal an irregularly shaped soft tissue mass with central areas of calcification and peripheral regions displaying hypodense tissue. These radiological features, while not pathognomonic, can raise suspicion and prompt further investigation for a potential diagnosis of EMCS.^9^, ^10^ For an accurate diagnosis of EMCS based on these findings, histopathologic tissue techniques are necessary. Histopathological examination of the tissue specimen obtained through biopsy or surgical excision allows for a definitive assessment of the neoplastic cells and their distinct characteristics, confirming the presence of EMCS and distinguishing it from other potential differential diagnoses. This crucial histopathologic evaluation plays a pivotal role in guiding appropriate management strategies and optimizing patient outcomes.^2^

We studied a 38‐year‐old female with EMCS, characterized by poorly differentiated round cells mixed with well‐differentiated hyaline cartilage. This rarity in the extraskeletal region and diagnostic challenges make this case unique, particularly in distinguishing it from other soft tissue cancers.

## 2 CASE PRESENTATION

**FIGURE 1:**
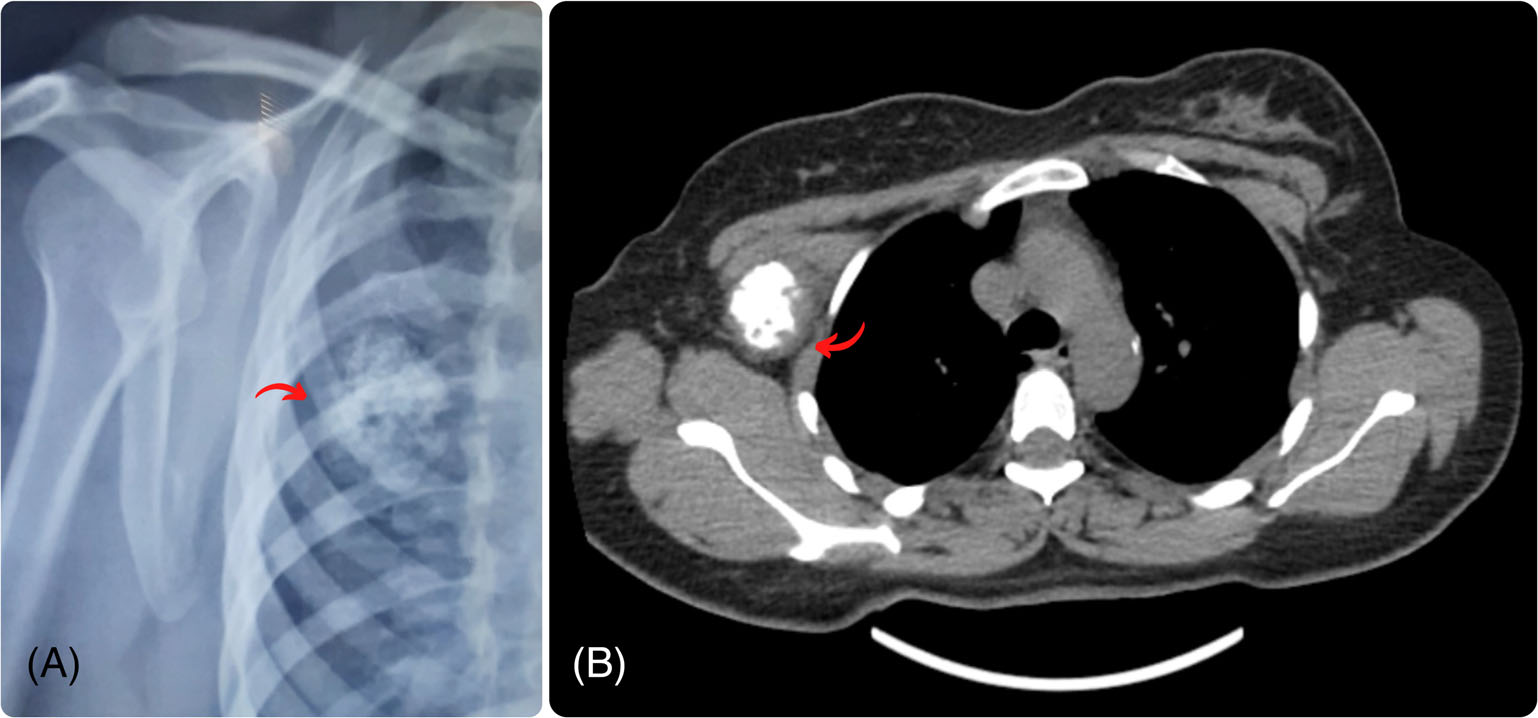
Primary soft tissue chest wall mesenchymal chondrosarcoma with biphasic pattern in a 38‐year‐old woman. (A) Axial non‐contrast CT shows a mass (arrow) in the right hemithorax under pectoralis muscle and inseparable from the second rib with a distinct calcified (central) and non‐calcified (periphery) components. (B) The chest X‐ray determined a well‐defined mass with intralesional rings and arcs calcification which is the near ribs.

**FIGURE 2:**
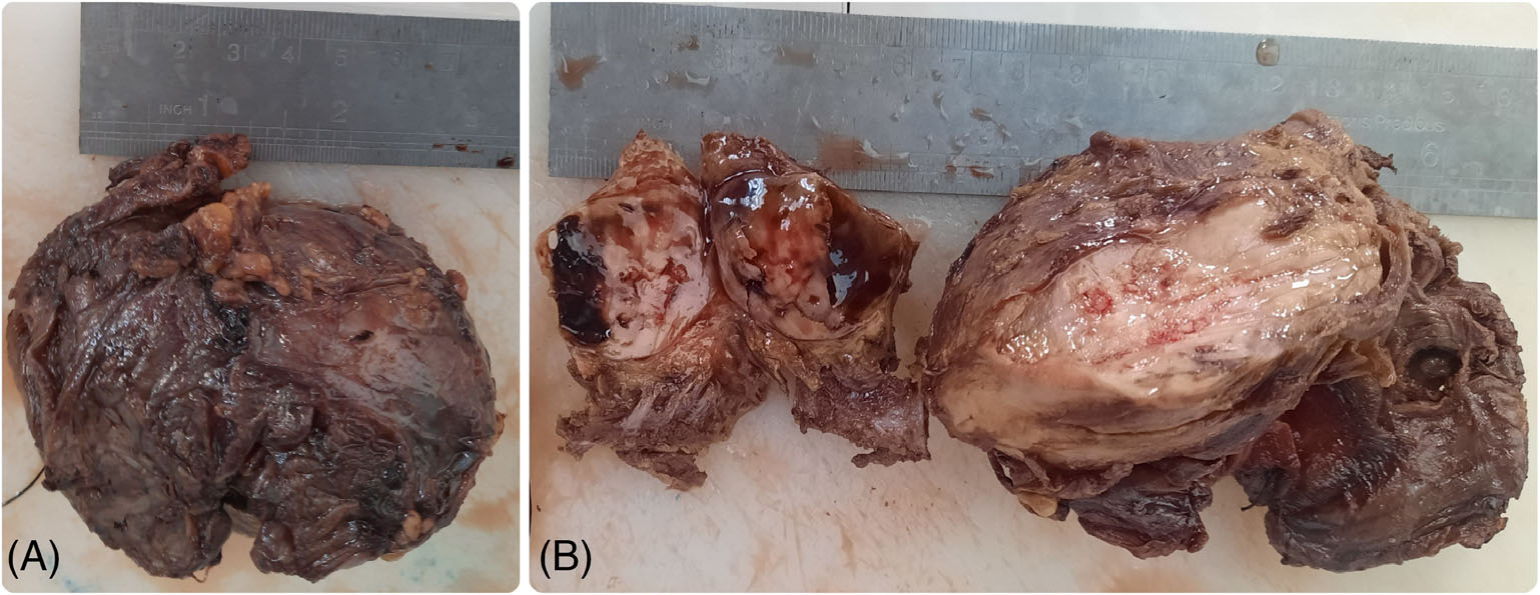
Gross images of resected specimen. (A) The tumor is tightly wrapped and seems to have a firm, flexible consistency. (B) The longitudinal section of the excised mass reveals a solid and fleshy appearance with focal cystic change.

**FIGURE 3:**
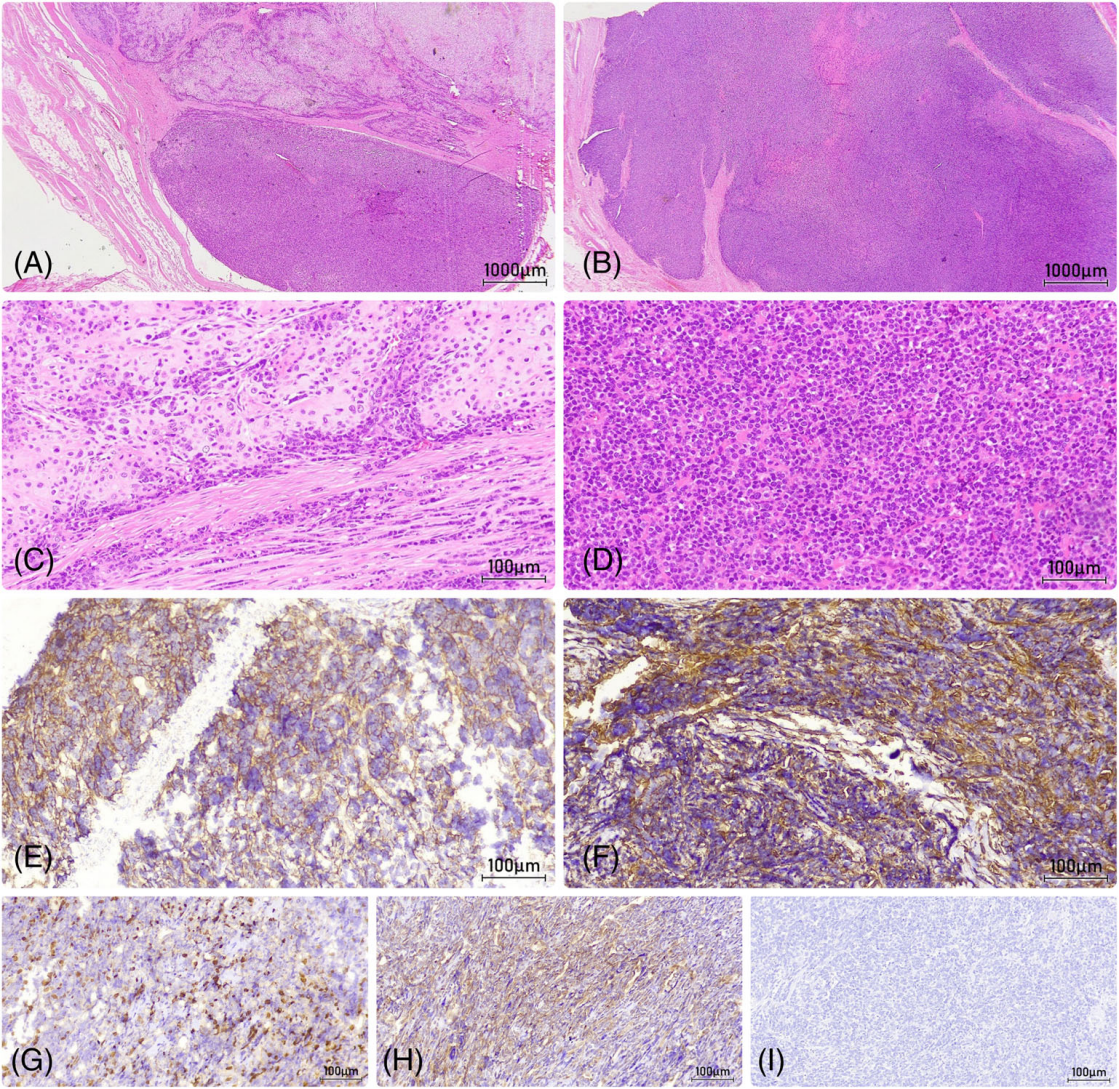
(A) H&E ×20: Malignant biphasic mesenchymal neoplasm with a well differentiated hyaline cartilage component (abrupt transition). (B) H&E ×20: Solid sheets of undifferentiated small blue cells. (C) H&E ×200: Solid sheets and strands of undifferentiated small blue cells mixed with islands of mature appearing, well differentiated hyaline cartilage. (D) H&E ×200: Cellular sheets of undifferentiated small blue cells with frequent mitotic activity. (E) IHC, ×200: CD99, membranous staining in tumor cells. (F) IHC, ×200: Vimentin, positive staining in tumor cells. (G) IHC, ×200: Ki67, high proliferative index. (H) IHC, ×200: NSE, positive staining in some tumor cells. (I) IHC, ×200: FLi1, negative.

In September 2022, a 38‐year‐old female with no significant medical background was referred to a radiologist by a midwife, owing to a 2‐month history of pain in the right upper quadrant of her right breast. The presence of the palpable mass had been noted 1 week before her initial visit. The medical assessment of the patient was subsequently conducted at Firooz Abadi Hospital. The sonography unveiled a calcified round mass with a soft tissue rim adjacent to the second rib, located beneath the major pectoralis muscle, with no apparent involvement of the right breast. The observed punctuated and matured calcification led to considering rib chondroma as a more plausible differentiated diagnosis. The chest X‐ray revealed the presence of a calcified round mass, potentially associated with the nearby rib (Figure 1A). The Computed tomography scan (CT‐scan) demonstrated a 72 × 50 × 30 mm mass located in the soft tissue of the right pectoral muscle, showing no invasion of the proximal rib bone and no presence of calcified lymph nodes in the vicinity. Moreover, the mass exhibited a fade rim enhancement, particularly in its peripheral part (Figure 1B). Histopathologic examination of the core‐needle biopsy (CNB) unveiled a malignant neoplasm characterized by a bimorphic pattern. This included sheets of undifferentiated round‐to‐oval cells with an abrupt transition to nodules of well‐differentiated cartilage. The tumor cells displayed large hyperchromatic and overlapped nuclei with scant cytoplasm. The hematological and biochemical analyses of the patient's blood specimen yielded no abnormal findings, After the surgical excision of the tumor, the gross view of the mass revealed lobulated contours with a solid, fleshy appearance, accompanied by focal cystic changes (Figure 2A,B). The histopathologic findings of the completely excised lesion were consistent with the initial CNB pathology report, revealing the presence of dilated vascular spaces (HPC‐like pattern) and foci of bone formation (Figure 3A). Based on the pathology interpretation, these characteristics were indicative of small round blue cell tumors and EMCS (Figure 3B,C). An immunohistochemical (IHC) study corroborated the diagnosis and effectively excluded other less probable histopathologic differential diagnoses, including Extraskeletal Ewing sarcoma. In the IHC study, the tumor cells exhibited positive expression for Bcl2, Ki67 (30%–40% tumor cells), CD99, NSE, and Vimentin, while FLi1 demonstrated negative staining (Table 1). As a result, the final diagnosis was determined to be consistent with EMCS.

**TABLE 1:** Immunohistochemical report. CD99 is positive but FLI1 and S100 is negative in tumor cells.

IHC marker	Result
CD99	Positive in most tumor cells
NSE^a^	Positive in some tumor cells
Chromogranin	Negative
Synaptophysin	Negative
S100	• Negative in tumor cell
	• Occasionally Positive in cartilaginous component
SMA^b^	Negative
Vimentin	Positive in most tumor cells
CD34	• Negative in tumor cell
	• Highlights vascular spaces
BCL2	Positive in most tumor cells
FLI1	Positive in some endothelial stromal cells
CD45	Negative
PanCK	Negative
EMA^c^	Negative
CK7	Negative
CK20	Negative
WT1	Negative
Ki67	Positive in about 30%–40% of tumor cells

^a^ Neuron‐specific enolase.

^b^ Smooth muscle actin.

^c^ Epithelial membrane antigen.

Following the successful excision of the mass, the patient was referred to a reference cancer center for a radiation oncologist consultation. Because the tumor's margins were negative and no malignant cells were seen, radiotherapy was not indicated. Soon after the patient received a 6‐month course of the VDC/IE‐regimen, which involved alternating drug combinations every 3 weeks. The treatment protocol consisted of two alternating combinations: (1) Ifosfamide at a dose of 17.5 g and Etoposide at a dose of 950 mg, and (2) Vincristine at a dose of 2 mg, Doxorubicin at a dose of 120 mg, and Cyclophosphamide at a dose of 2200 mg. This treatment regimen was administered for a period of 6 months, with careful monitoring and documentation of the patient's response to the therapy. Throughout the 6‐month chemotherapy period and after its completion, the medical team closely observed and revisited the patient to monitor her response to treatment and manage any potential side effects. At the 10‐month mark, the patient remains asymptomatic with no tumor recurrence or complications from the surgery or chemotherapy. Her overall health is excellent, and she can perform routine tasks without any limitations.

## 3 DISCUSSION

The key finding in diagnosing this particular case was the identification of a biphasic morphology within the tumor. The initial CT scan revealed the presence of a fade rim enhancement in the mass, particularly in its periphery. In our case, the presence of a distinct demarcation between soft tissue and calcification aroused the suspicion of MCS among the medical team. With this suspicion, the CNM was done to ensure that the sampling method was appropriate and that the removed sample had soft and calcified tissue. The histopathological investigations have verified the presence of a well‐differentiated hyaline cartilage component alongside solid sheets of undifferentiated small blue cells that display a clear border between the two components.

Bimorphic pattern is crucial because if it is not considered during sampling with fine‐needle biopsy or core‐needle biopsy and the sample does not contain cartilage tissue, it puts small blue cell tumors such as Ewing's sarcoma at the top of the differential diagnosis list. The matter becomes increasingly ambiguous given that specific markers, such as CD99, exhibit positivity in both MCS and Ewing sarcoma during standard IHC tests.

The presence of MCS in extraosseous tissues makes this case more unique and makes the diagnosis more challenging. Although MCS had previously been documented, for the first time, MCS was discovered in extra‐osseous tissues in 1964. However, due to its rarity, it remains a diagnostic and treatment challenge.^11^ EMCS has been reported in 14%–73% of MCS cases, with the largest series reporting 39% having extraskeletal tumors.^12^, ^13^ A malignant soft tissue tumor called EMCS that arises from chondroprogenitor cells is exceptionally uncommon. It is believed that primitive mesenchyme cartilage is the origin of differentiated cartilage as well as undifferentiated round or spindled cells.^14^

In practice, all patients with an unexplained deep soft tissue mass or with a superficial soft tissue lesion with a diameter of more than 5 cm should be referred.^15^

In light of this case, it is also important to remember that not all masses felt deeply during a breast examination are made up of breast tissue. Clinical characteristics of breast and chest wall lesions may be mistaken. Breast sonography can examine a chest wall lesion that appears as a breast mass at the start of the evaluation. Sonography is increasingly being utilized to evaluate palpable superficial chest wall masses.^16^

Although ultrasound is utilized to examine tumors in the chest wall, it should be clarified that mammography is the preferred and standard breast cancer screening method and is recommended for women beginning at age 40 years.^17^ Breast ultrasonography, utilized in high‐resource settings to supplement mammography in some clinical circumstances, presents a potentially feasible option for early diagnosis of breast cancer in some resource‐limited regions since it is portable, less expensive than mammography, and valuable across a larger variety of clinical applications.^18^ Even though our patient visited the midwife with the complaint of a palpable mass in her breast, and some references state that women her age who have this complaint can undergo mammography as an initial step, the midwife ordered ultrasonography. The mass was discovered to be outside the breast tissue during sonography. Her mammogram was later found to be normal. Because the mass was at a high‐level location, it was not visible on the mammogram.

Standard radiographs are one of the modalities used in the diagnosis. They can rule out a bone tumor, detect bone erosion that could lead to fracture, and show calcifications. Although ultrasound seems to be the initial exam for chest wall lesions, it should be followed by CT or MRI. Magnetic resonance imaging (MRI) is considered the primary imaging modality for primary soft tissue tumors. However, CT scans are also a viable option for imaging these types of tumors.^15^ Our patient underwent a CT scan because the ultrasound revealed a calcified mass.

The utilization of MRI was deemed suboptimal in this case due to the creation of artifacts, particularly in light of the mass's calcification. CT was therefore favored over MRI. Although the CT scan identified biphasic morphology as a diagnostic clue, the MRI imaging did not yield any supplementary information. Due to the lack of specificity in the data, MRI was not done in the current case.

The presence of a precisely defined soft tissue mass exhibiting granular, ring‐and‐arc, or irregularly shaped calcifications may be detected on a CT scan of MCS tumors. The non‐mineralized components of the neoplasm exhibit a lower density in comparison to the adjacent soft tissue. A biphasic morphology with a clearly distinguishable calcified and noncalcified tumor component is observed in approximately one‐third of cases of MCS on imaging.^14^, ^19^ The current case was among those cases that demonstrated a hypodense periphery and a biphasic appearance.

The MRI images support the prior findings. Based on the research done on MCS, the hypointense signal can be seen on T1W images. Lesions appear more intense on T2W images than skeletal muscle. The more calcified masses appeared more heterogeneous than, the less calcified masses.^19^

Given that the imaging characteristics lack specificity for EMCS and have been demonstrated in other non‐malignant cartilaginous tumors, an accurate diagnosis might require histopathological tissue sampling through a range of techniques such as fine‐needle aspiration, core‐needle biopsy, incisional biopsy, and excisional biopsy.^14^, ^19^

The histology of EMCS is bimorphic and consists of islands of hyaline cartilage and small round cell regions. Many tumors show a noticeable pericytomatous vascular pattern. Most EMCS comprises tightly spaced small cells with specific cytologic characteristics, such as round or oval nuclei with dense, coarse chromatin and scant, indistinct cytoplasm. Endochondral ossification is frequently observed in EMCS and is occasionally mistaken as osteosarcoma. A multinodular pattern can be formed when several small islands of hyaline cartilage combine with small round cells. The cells in some EMCS tumors are fascicle‐arranged and spindle‐shaped. Even though spindle cell regions often make up a small percentage of a particular tumor. In rare cases, the fibrotic stroma is also seen.^20^ The present study reveals the existence of a well‐differentiated hyaline cartilage component and solid sheets of undifferentiated small blue cells, which is in accordance with the current knowledge on the pathology of MCS.

MCS has a remarkable biphasic pattern, and the diagnosis is simple if both portions are collected. Nevertheless, the absence of cartilage in the biopsy sample might lead to confusion with other small rounds, blue‐cell tumors, such as Ewing's sarcoma, lymphoma, neuroblastoma, small‐cell osteosarcoma, and desmoplastic small round cell tumor.^21^ Both mature cartilage cells and round cells were found in the biopsy of the case being discussed, indicating a biphasic pattern. Having both tissues simplified the diagnosis for us, but the tissue may only represent the tumor's round cell component; in these cases, further workup is required for accurate interpretation.^22^ When a proper biopsy is unavailable, practitioners may consider getting larger biopsy samples to reduce sampling error or verify the diagnosis using immunomarkers or genetic analysis.

In immunohistochemical analysis, positive staining for CD99 and SOX9 was seen in MCS tumor cells. The protein Sox9 can serve as a discriminative marker to differentiate MCS from other small blue round cell tumors. S100 protein is exclusively expressed in cartilage in MCS, and EMA is positive in some cases. In our current study, the EMA was negative.^2^, ^14^, ^23^, ^24^ FLI1 is negative in MCS, which helps distinguish it from Ewing's sarcoma.^25^ Besides the biphasic pattern, the absence of the FLI‐1 marker in the tumor cells from the current case helped us rule out the possibility of Ewing sarcoma. The results indicate negative SMA, CD45, and keratin expression in the MCS tumors.^26^, ^27^

Furthermore, genetic testing can be used to avoid diagnostic errors in cases where the available sample size is inadequate. The presence of a recurrent HEY1‐NCOA2 rearrangement has the potential to be identified in cases of MCS. The previously mentioned fusion has been observed in nearly all extensively studied MCSs, while its presence is not detected in other CS subtypes.^2^

Since our patient's MCS tumor was found in the axial region, we looked at the previously documented differences in survival rates between individuals with tumors in the axial and non‐axial regions. When considering all sites, the prognosis for MCS is usually poor.^4^ Overall survival for MCS was 51% at 5 years and 43% at 10 years, according to Schneiderman et al, the review on MCS, with no difference in survival between extraskeletal and skeletal malignancies. Moreover, axial EMCS tends to have worse overall survival when compared to appendicular and cranial areas.^12^ According to a review study on both skeletal and non‐skeletal MCS patients by Xu, Jie, et al, the 5‐ and 10‐year overall survival rates for the axial area are 41.0% and 22.8%, respectively. However, they are 55.3% and 44.2%, respectively, for non‐axial regions. They suggested that the complexity of attaining broad surgical margins in the axial area might explain this difference.^28^

In the end, it is noteworthy to mention the treatment of MCS. The role of surgery in the local treatment of MCS is crucial.^29^ Cesari et al reported that patients who underwent wide‐ranging surgical resection had a better likelihood of survival. All patients not surgically disease‐free died after a median of 22 months, demonstrating the need for surgery in MCS therapy.^30^ Although chemotherapy's role in treating MCS is unclear, Frezza's study showed that administering chemotherapy to patients with localized disease was associated with a decreased risk of mortality and recurrence.^5^ Our patient was treated with the VDC/IE‐regimen chemotherapy following the removal of the tumor, and she has been symptom‐free up to the time of this report's preparation.

## 4 CONCLUSION

In conclusion, the diagnosis of EMCS poses a significant challenge due to its rarity and occurrence in various extra‐skeletal tissues. Accurate diagnosis of MCS is crucial for effective tumor management. Therefore, physicians must carefully assess the biphasic pattern of the masses in imaging studies. Inadequate specimens may hinder the detection of the characteristic bimorphic pattern of MCS during pathological assessment, where both cartilage and small blue cells coexist. Completion of comprehensive diagnostic workups, including performing immunohistochemistry (IHC) tests, is essential to rule out other potential small blue cell tumors and arrive at an accurate diagnosis.

## AUTHOR CONTRIBUTIONS

**Razieh Shahnazari:** Conceptualization (equal); data curation (equal); writing – review and editing (equal). **Fatemeh Montazer:** Conceptualization (equal); resources (equal); validation (equal). **Shahriar Shirzadi:** Validation (equal); writing – original draft (equal); writing – review and editing (equal). **Sina Karaji:** Visualization (equal); writing – original draft (equal); writing – review and editing (equal).

## CONFLICT OF INTEREST STATEMENT

The authors have stated explicitly that there are no conflicts of interest in connection with this article.

### ETHICS STATEMENT

The patient provided written informed consent for the publication of this case report and accompanying images.

## Data Availability

The data that support the findings of this study are available on request from the corresponding author. The data are not publicly available due to privacy or ethical restrictions.
